# A novel prognostic model to predict outcome of artificial liver support system treatment

**DOI:** 10.1038/s41598-021-87055-8

**Published:** 2021-04-05

**Authors:** Jin Shang, Mengqiao Wang, Qin Wen, Yuanji Ma, Fang Chen, Yan Xu, Chang-Hai Liu, Lang Bai, Hong Tang

**Affiliations:** 1 Center of Infectious Diseases, West China Hospital, Sichuan University, No. 37 Guoxue Alley, Wuhou District, Chengdu, 610041 Sichuan China; 2 Department of Epidemiology and Biostatistics, West China School of Public Health and West China Fourth Hospital, Sichuan University, Chengdu, Sichuan China

**Keywords:** Infectious diseases, Hepatology, Outcomes research

## Abstract

The prognosis of Artificial liver support system (ALSS) for hepatitis B virus-related acute-on-chronic liver failure (HBV-ACLF) is hard to be expected, which results in multiple operations of ALSS and excessive consumption of plasma, increase in clinical cost. A total of 375 HBV-ACLF patients receiving ALSS treatment were randomly divided a train set and an independent test set. Logistic regression analysis was conducted and a decision tree was built based on 3-month survival as outcome. The ratio of total bilirubin before and after the first time of ALSS treatment was the most significant prognostic factor, we named it RPTB. Further, a decision tree based on the multivariate logistic regression model using CTP score and the RPTB was built, dividing patients into 3 main groups such as favorable prognosis group, moderate prognosis group and poor prognosis group. A clearly-presented and easily-understood decision tree was built with a good predictive value of prognosis in HBV-related ACLF patients after first-time ALSS treatment. It will help maximal the therapeutic value of ALSS treatment and may play an important role in organ allocation for liver transplantation in the future.

## Introduction

Acute-on-chronic liver failure is a clinical syndrome characterized by an acute deterioration of pre-existing liver disease in both cirrhotic and noncirrhotic patients who will develop multiple-organ failure with high short-term mortality. There are two different definitions proposed by Asia Pacific Association for Study of Liver ACLF Research Consortium (AARC)^1^ and European Association for Study of Liver-Chronic liver Failure Consortium (EASL-CLIF)^2^ because of different main etiology of areas^3^. In China, HBV is exactly the most common cause and HBV-related ACLF accounts for about 70% of the total ACLF patients^4^. Compared with other causes of ACLF, HBV-related ACLF exhibits higher rates of bacterial and fungal infections and lower rate of renal failure with higher mortality^5^. Treatment of HBV-ACLF patients include general management, specific treatment, bridging therapies, and liver transplantation. Whether general management plus antiviral strategies could improve the prognosis is controversial^6,7^. Liver transplantation is proved to be the most effective therapy with 1-year survival rate reaching 87%^8,9^, but the difficulties in urgent transplantation assessment and lack of donors limit its application.

Therefore, the artificial liver support system (ALSS), a kind of extracorporeal support therapy, plays an essential role in the management of ACLF. Although whether ALSS could improve the prognosis of HBV-ACLF patients is controversial. Two large randomized multicenter controlled trails showed no benefit of ALSS for ACLF^10,11^, however, a meta-analysis including 13 random control trails and another systematic review revealed 74 studies including 17 RCTS showed improved survival in ACLF with ALSS treatment^12,13^. Heterogenous group of patients is part of the reason of this difference. On the other hand, the pattern of ALSS treatment is also improved from plasma exchange (PE) or molecular adsorbent circulating system (MARS) to double plasma molecular adsorbent circulating system (DPMARS), which has common advantages and lower adverse events compared to PE and MARS. Recent reaches showed DPMARS improved the short-term survival of ACLF^14,15^. However, the clinical challenge is that, the benefit of the ALSS treatment is hard to expect, thus patients with HBV-related ACLF generally need three to five times of ALSS operations. Even though some patients need more than ten times of ALSS operations, severity and deterioration of liver diseases still occur (15–50%). Therefore, a predictive model is needed to maximal the therapeutic value of ALSS treatment and help decide the timing of continuing ALSS treatment or urgently needing LT, also large amount of social resources could be saved.

Previously, a variety of models were reported to predict the prognosis of ACLF patients including MELD score, MELD-Na, CTP, CLIF-C ACLF score, SOFA, APACHE II, and AARC)^16–18^. Among these, the MELD score and CTP are the most commonly used (clinically) and form the basis of organ allocation for liver transplantation. Moreover, recent studies indicated that dynamic models rather than the models based on the characteristics at attendance have better predictive value on the prognosis of ACLF^19^. However, no consensus exists regarding how to assess the benefit of ALSS treatment.

This study was designed to explore a model based on using baseline parameters or dynamic parameters to predict the short-term prognosis of patients with HBV-related ACLF. The aim of this study was to address the issue whether patients could benefit from ALSS treatment or need urgent liver transplantation, which could help maximal the ALSS benefit and decide the organ allocation.

## Results

### Study population

A total of 375 HBV-ACLF patients who underwent ALSS treatment were included from 2014 to 2017 in the single hospital (annual count: 94 in 2014, 99 in 2015, 89 in 2016, and 93 in 2017). Among them 337 (89.9%) were males. Age distribution was mainly unimodal with a peak in the 40–49 years-old interval (Figure S1A). Then we applied machine learning by randomly dividing the 375-patient cohort into a train set (n = 282, 75% of the sample) and an independent test set (n = 93, the remaining 25% of the sample). The train set was used for building a statistical and predictive model, and the test set was subjected to evaluate the robustness of model performance. Baseline parameters including age, gender, HBV DNA, total bilirubin, ALT, AST, creatinine, INR, cirrhosis rate, CTP score, MELD score, survival rate all showed no significant difference between train set and test train (Table 1).

**Table 1:** Baseline characteristics of HBV-related ACLF patients.

Variable	Train set (n = 282)	Test set (n = 93)	P value
Age	44.42 ± 10.59	42.61 ± 10.61	0.63
Men	90.78%	87.10%	0.307
CTP score	10.15	10.2	0.617
**CTP grade**			0.401
A	0	0	–
B	92	26	–
C	190	67	–
MELD score	26.51	27.14	0.822
Lg (HBV DNA)	4.79	4.86	0.221
Total bilirubin (mg/dL)	432.3	462.27	0.763
ALT (IU/mL)	317.58	333.53	0.119
AST (IU/mL)	235.15	250.67	0.208
Creatinine (mg/dL)	87.45	90.98	0.181
INR	2.24	2.23	0.262
Cirrhosis	0.83	0.78	0.373
Survival rate	0.62	0.62	0.994

Significance level: α = 0.05, *ALT* alanine transaminase, *AST* aspartate transaminase, *INR* international normalized ratio.

### Predictor for survival screening

Totally 234 patients were still alive and 141 patients were dead with overall 3 months survival rates as 62.4%. Sex composition and age distribution displayed no statistical difference between the two outcome subgroups (Figure S1B and S1C). Then, the panel of physiological parameters of both baseline level and the dynamic level, as well as established medical evaluations, were compared between the two subgroups (Table S1 and Figure S2).

Unconditional logistic regression, a form of generalized linear model, was applied to investigate the association of each parameter with the outcome, and some parameters displayed strong significance (Table 2) and some of the remaining parameters are significant at the significance level of 0.05 but not the more conservative adjusted level; therefore, their association with patient survival was likely less solid (Table S2).

**Table 2:** Candidate parameters significant in univariate logistic regression.

Parameter	*β*	95% CI for *β*	*P* value
**Baseline level**			
Creatinine	− 0.016	(− 0.026, − 0.008)	0.0004988*
log_10_(platelet)	2.769	(1.598, 4.014)	0.0000066*
**Dynamic level**			
Total bilirubin	− 0.024	(− 0.032, − 0.017)	0.0000000*
Direct bilirubin	− 0.017	(− 0.025, − 0.01)	0.0000034*
Total protein	− 0.123	(− 0.189, − 0.061)	0.0001413*
RPTB	− 14.655	(− 18.813, − 10.957)	0.0000000*
**Classic score**			
CTP grade	− 1.866	(− 2.583, − 1.224)	0.0000001*
CTP score	− 0.934	(− 1.195, − 0.702)	0.0000000*
MELD score	− 0.184	(− 0.258, − 0.117)	0.0000003*
MELD.Na score	− 0.113	(− 0.163, − 0.069)	0.0000021*

Significance level: α = 0.05; αadj = α/46 = 0.001, adjusting for hypothesis tests of 46 factors. *P value both < α and < αadj; Dynamic level: change level of parameters before and after first-time ALSS treatment;

*RPTB* Residual percentage of total bilirubin: total bilirubin post-first time ALSS next-day/ total bilirubin pre-first time ALSS.

Finally, the overlap match of hit parameters by both hypothesis test and statistical model revealed the most particular factors were dynamic change of total bilirubin named RPTB (residual percentage of total bilirubin: total bilirubin post-first time ALSS next-day/total bilirubin pre-first time ALSS), CTP grade, CTP score, MELD score, and MELD-Na score.

### Development of a decision tree

**Figure 1:**
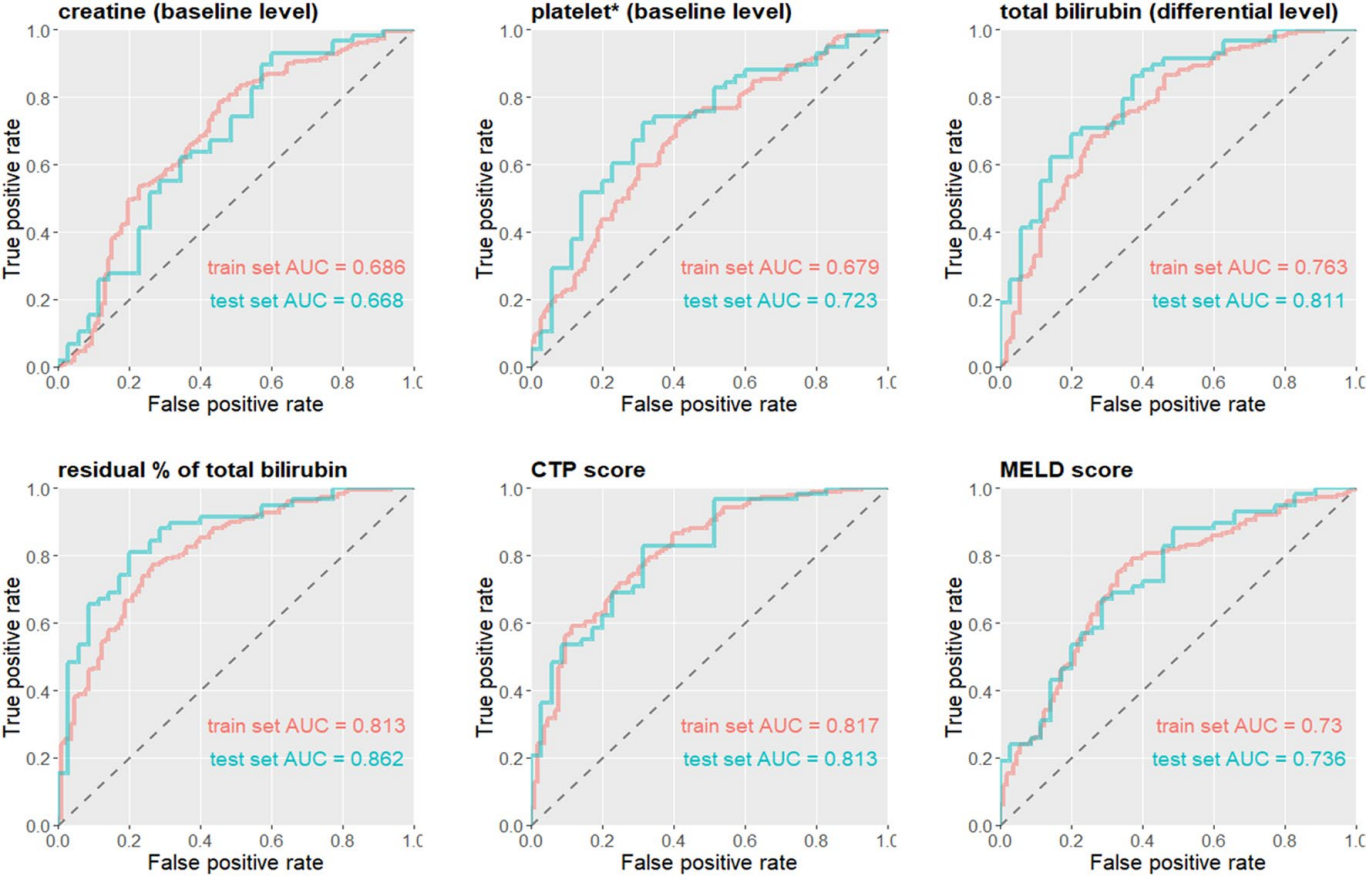
ROC (receiver-operation-characteristics) plots and AUC (area-under-curve) values of the train set and the test set for univariate analysis of significant factors. *Presented in *log*_*10*_ scale.

Logistic regression was applied for univariate analysis of each variable given the train set (Table 3). Model performance was judged in the test set. Presentation of binary classification result in the receiver-operation-characteristics (ROC) curve, and the associated area-under-curve (AUC) value help further validate the predictive effectiveness of the model (Fig. 1). Apparently, RPTB is the most significant factor (achieving accuracy of 0.796, F1-score of 0.829, and AUC of 0.862). For the classic score, CTP score behaves the most significant medical evaluation; there seems no apparent difference between MELD score and MELD-Na score in predicting ALSS survival.

**Table 3:** Model performance of selected parameters in the test set.

Parameter	Accuracy	Kappa	Sensitivity	Specificity	PPV	NPV	F1-score
**Baseline level**							
Creatine	0.645	0.231	0.741	0.486	0.705	0.531	0.723
log_10_(platelet)	0.699	0.373	0.724	0.657	0.778	0.590	0.750
**Dynamic level**							
Total bilirubin	0.731	0.431	0.776	0.657	0.789	0.639	0.783
Direct bilirubin	0.688	0.347	0.724	0.629	0.764	0.579	0.743
Total protein	0.688	0.316	0.793	0.514	0.730	0.600	0.760
RPTB	0.796	0.577	0.793	0.800	0.868	0.700	0.829
**Classic score**							
CTP grade	0.602	0.279	0.414	0.914	0.889	0.485	0.565
CTP score	0.720	0.418	0.741	0.686	0.796	0.615	0.768
MELD score	0.677	0.321	0.724	0.600	0.750	0.568	0.737
MELD.Na score	0.710	0.399	0.724	0.686	0.792	0.600	0.757
**Two-variate model**							
TB (d) + CTP	0.785	0.547	0.810	0.743	0.839	0.703	0.825
DB (d) + CTP	0.774	0.522	0.810	0.714	0.825	0.694	0.817
RPDB + CTP	0.785	0.542	0.828	0.714	0.828	0.714	0.828
TB (d) + MELD	0.763	0.518	0.741	0.800	0.860	0.651	0.796
DB (d) + MELD	0.731	0.449	0.724	0.743	0.824	0.619	0.771
RPDB + MELD	0.796	0.577	0.793	0.800	0.868	0.700	0.829
**Conditional inference model**							
Decision tree	0.796	0.567	0.828	0.743	0.842	0.722	0.835

Dynamic level: change level of parameters before and after first-time ALSS treatment; RPTB: residual percentage of total bilirubin, total bilirubin post-first time ALSS next-day/total bilirubin pre-first time ALSS; TB(d): difference level of TB: TB post-ALSS minus TB pre-ALSS. DB(d): difference level of DB, DB post-ALSS minus DB pre-ALSS. PPV: positive predictive value; NPV: negative predictive value.

**Figure 2:**
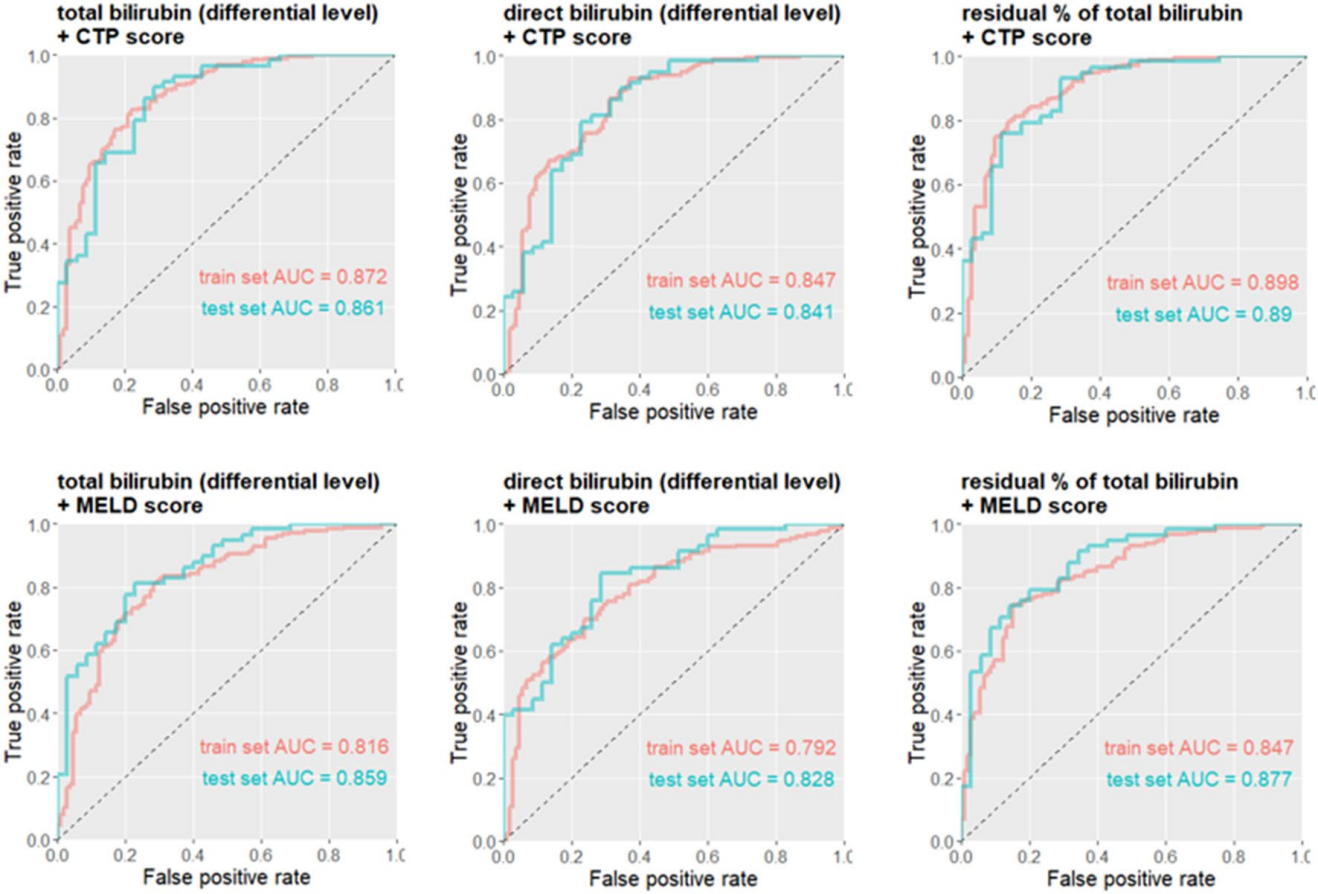
ROC plots and AUC values of the train set and the test set for multivariate analysis of selected combinations of significant factors.

**Figure 3:**
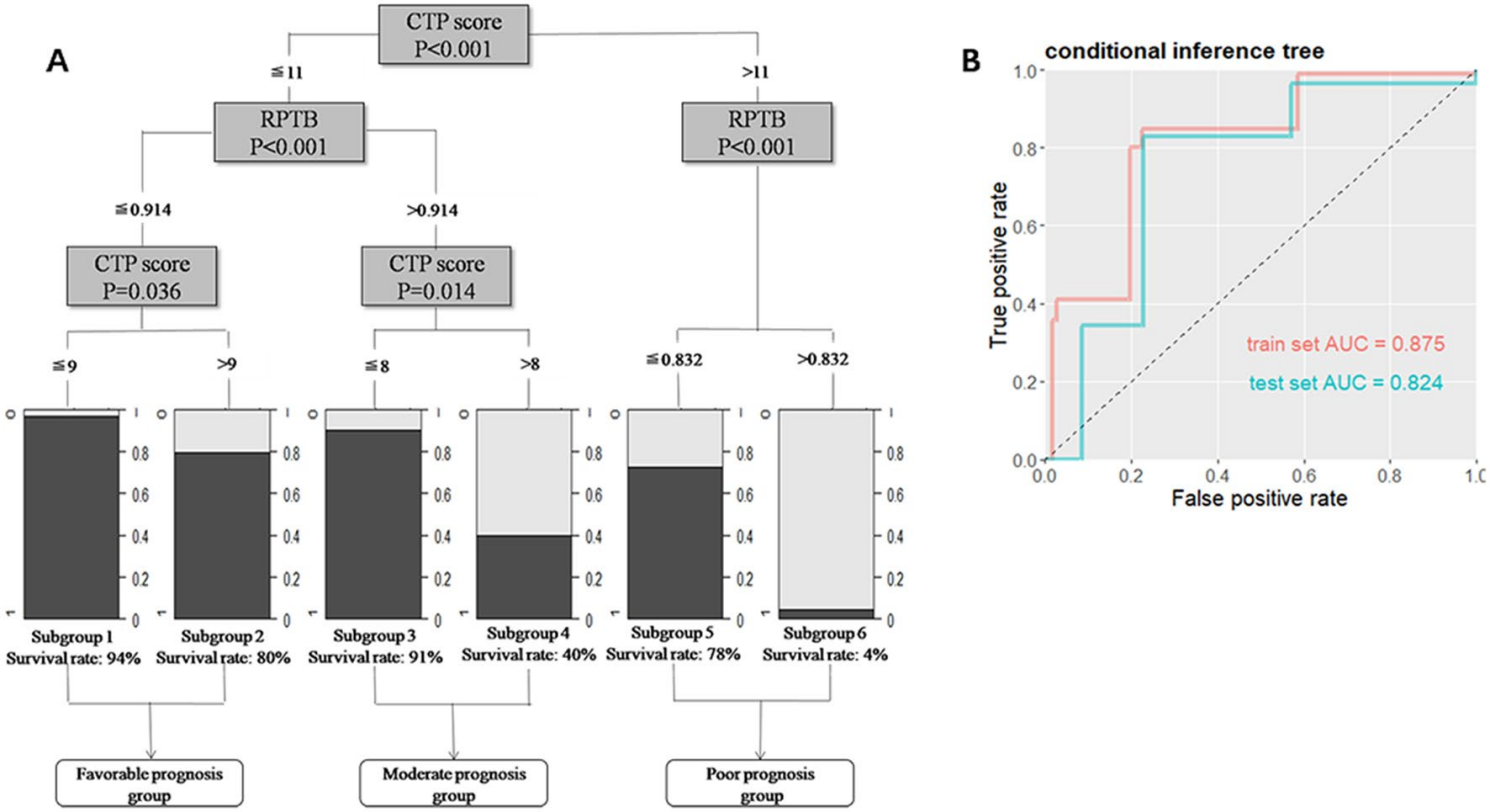
Conditional inference tree model for prediction of 3-month survival in ALSS-treated HBV-ACLF patients. (**A**) The decision tree; (**B**) ROC plot and AUC value for the tree model.

We further extended the analysis into multivariate logistic regression model and compared a selected combination of hit factors (Fig. 2). Best predictive performance was reported when RPTB was combined with CTP score and slightly lower with MELD score (Table 3). Finally, we adopted the method of decision tree to explore and validate the association of key factors with post-ALSS treatment, as this bypassed the complex “black-box” machine learning model. A two-layer tree structure was constructed with hit factors of CTP score and residual percentage of total bilirubin (Fig. 3).

Totally, patients were divided into 3 main group: favorable prognosis group, moderate prognosis group and poor prognosis group according to short-term survival. In favorable prognosis group, patients had both low CTP score (≤ 11) and low RPTB (≤ 0.914), the two sets of patients in favorable group both had high survival rate (94% and 80%). In moderate prognosis group, patients had low CTP score (≤ 11) but high RPTB (> 0.914), patients with higher CTP score (> 8 and ≤ 11) showed significant low survival rate as 40%, patients with lower CTP score (≤ 8) showed higher survival rate as 91%. While in the poor prognosis group, patients had high CTP score (> 11), patients with higher RPTB (> 0.832) showed lowest survival rate as 4%, only in patients with pretty low RPTB (≤ 0.832), survival rate could reach to 78%. The conditional inference tree model was validated on the test set (Table 3) and this nonlinear tree model performed similarly well as the linear logistic model reported above. Linear and nonlinear statistical models that were mutually compatible and robustly predictive of post-ALSS survival in HBV-ACLF patients.

## Discussion

The present study analyzed the clinical course of HBV-related ACLF receiving ALSS treatment. The residual percentage of total bilirubin (RPTB), which means the ratio of total bilirubin post-first time ALSS next-day to total bilirubin pre-first time ALSS, was the most significant factor associated with prognosis through logistic regression for univariate analysis. Second, a clearly presented and easily understood decision tree based on CTP and RPTB logistic regression model was made. Patients were divided into three main groups such as favorable prognosis group, moderate prognosis group and poor prognosis group. Patients had both low CTP score and low RPTB had favorable prognosis. While patients had high CTP sore or high RPTB showed lower survival rate. Patients who could benefit from continuing ALSS treatment or urgently awaiting liver transplantation could be distinguished according to this decision tree.

The balance of necrosis and regeneration of liver cells could determine the prognosis of patients with HBV-related ACLF. During the ALSS treatment, ALSS could first rapidly and significantly reduce the level of total bilirubin right after ALSS treatment. Then, the total bilirubin will rebound because of liver cell necrosis, cholestasis and bilirubin accumulation in the bile capillary or tissue space, thus a new regeneration environment was proved by ALSS treatment. In this study, we found that RPTB could reflect the regeneration ability of live with the help of ALSS, while CTP could reflect the necrosis of liver. This is why the decision tree based on combination of these two parameters could showed good predict value.

As the result of unconditional logistic regression, RPTB was the most significantly predictive factor. In addition, other parameters which displayed significance like baseline creatine and platelet, CTP score and MELD score were also reported to be associated with the prognosis of ACLF in previous reports^20,21^. Three key factors influencing the efficacy of ALSS treatment. First, total bilirubin at baseline reflects the severity of underlying liver injury. Second, rebound of total bilirubin after ALSS treatment reflect the obstruction of bile capillary and pressure of total bilirubin in tissue space^22^. Third, the level of total bilirubin next day after ALSS treatment reflects the new vascular homeostasis and regeneration circumstance. Therefore, compared with baseline total bilirubin, change of total bilirubin, and the rebound of bilirubin, etc. only the RPTB could reflect the baseline level of severity of liver injury, the rebound of bilirubin, and the new regeneration environment achieved by first time ALSS treatment. If patients had low RPTB, it means that ALSS treatment effectively removes toxic substance with a lower rebound rate, which results in an appropriate environment for liver regeneration and spontaneous recovery during the acute exacerbation period; therefore, the prognosis is predicted to be good.

In regard to CTP score which showed better predictive value than MELD score. CTP score included grade of encephalopathy, ascites, PT, total bilirubin, and albumin which is more suitable to the ACLF defined by APASL. On the other hand, according to the characteristics reported in a previous research, eastern-type ACLF exhibits relatively lower rates of renal failure, respiratory failure, circulatory failure but higher rates of coagulation abnormalities, ascites, and encephalopathy^23^. CTP score is more effective to assess the liver and coagulation failure and complications in eastern-type ACLF. Therefore, CTP score manifested better predictive value than MELD score in assessing the efficacy of ALSS treatment in HBV-related ACLF. While the western definition of ACLF considers organ failure as an essential part of the definition, MELD score showed better predictive value in western-type ACLF patients.

With respect to the application value of decision tree, in the CANONIC study, among the patients, only 9% patients with ACLF underwent liver transplantation within 28 days after admission^3^ and a large proportion of patients died while waiting for surgery. Organ allocation for ACLF is essential and patients with MELD score over 35 were reported to be superior to receive LT in a previous research^24^. However, in this study, MELD score didn’t show best predict value of prognosis of HBV-ACLF patients with ALSS treatment. Although the benefit of ALSS for patients with ACLF remain controversial, the decision tree could contribute to sort out patients need urgently receiving liver transplantation or continuing ALSS treatment our decision tree, 3 main groups were divided including favorable prognosis group (survival rate > 80%), moderate prognosis group (60–80%) and poor prognosis group (survival rate < 60%). In poor prognosis group, continuing ALSS could act as a bridge therapy to remove bilirubin and other toxicities and supply albumin and coagulation factors to reduce the occurrence complication such as hepatic encephalopathy, patients hence acquire more stable liver status when waiting for liver transplantation. While in favorable prognosis group, continuing ALSS treatment could not only successfully remove toxicities but also provide a better liver regeneration circumstance to improve the prognosis of HBV-ACLF. The well-divided predict model could maximal the therapeutic value of ALSS treatment and help decide the timing of continuing ALSS treatment or urgently needing Liver Transplantation. The applied value of decision tree for organ allocation is therefore worth exploring in the future study.

## Conclusion

The dynamic parameter RPTB was found to be strongly associated with the prognosis of patients with HBV-related ACLF receiving ALSS treatment. Moreover, decision tree based on predictive model combining RPTB and CTP score could divide patients into 3 group with significant different prognosis. Which may play an important role in maximal the therapeutic value of ALSS treatment and help decide the organ allocation for liver transplantation in the future.

## Methods

The study protocol was approved by the ethic committee of West China Hospital of Sichuan University and conformed to the ethical guidelines of the 1975 Declaration of Helsinki, informed consent was obtained from each patient or his/her legal guardian. We evaluated patients 18 or older listed for artificial liver support system from 2014 to 2017. The included patients with ACLF were based on APASL definition^1^. The definition is : ACLF is an acute hepatic insult manifesting as jaundice (serum bilirubin ≥ 5 mg/dL (85 µmol/L) and coagulopathy (INR ≥ 1.5 or prothrombin activity < 40%) complicated within 4 weeks by clinical ascites and/or encephalopathy in a patient with previously diagnosed or undiagnosed chronic liver disease/cirrhosis, and is associated with a high 28-day mortality. The inclusion criteria were: (1) Jaundice [serum bilirubin ≥ 5 mg/dL (≥ 85 µmol/L)]; (2) Coagulopathy (international normalized ratio [INR] ≥ 1.5 or prothrombin activity ≤ 40%); and (3) Any degree of encephalopathy and/or clinical ascites within 4 weeks on the basis of ongoing chronic liver diseases. (4) HBsAg positivity for more than 6 months. All the patients received double plasma molecular adsorption system (DPMARS) plus plasma exchange (PE) as ALSS treatment. For model building and validation, patients were randomly segregated into mutually exclusive and collectively exhaustive train set and test set in 3:1 ratio.

Patients were assayed for a panel of three sets of key parameters. Parameters at admission as the baseline level and classical model for ACLF patients, e.g. Child-Turcotte-Pugh (CTP) score and Model for End-stage Liver Disease (MELD) score were compared. To explore the predict value of dynamic parameters, change level of parameters before and after first-time ALSS treatment were also evaluated. Measured physiological parameters included total bilirubin, albumin etc. In addition, were calculated for individuals. All of the above variables together served as the potential factors-of-interest. All patients were followed up and the outcome of survival vs. death by 3 months post-ALSS was the major endpoint event of interest. All authors had access to the study data and reviewed and approved the final manuscript.

## Data analysis

All data analysis was conducted in version 3.4.2 of the R statistical environment (R core team, 2017). Logistic regression model and conditional inference tree model were built using the *glm()* function of *base* package and the *ctree()* function of the *party* package. Potential confounders such as sex and age were included as covariates in the statistical models. Significance level of 0.05 was used for hypothesis tests, and Bonferroni was adjusted for multiple tests.

## Supplementary Information

Supplementary Information.

Supplementary Figure S1.

Supplementary Figure S2.

Supplementary Figure S3.

Supplementary Table S1.

Supplementary Table S2.

## Abbreviations

ACLF: Acute on chronic liver failure
HBV: Hepatitis B virus
ALSS: Artificial liver support system
CTP: Child-Turcotte-Pugh
MELD: Model for end stage liver disease
