# Supplementary material for: A novel prognostic model to predict outcome of artificial liver support system treatment
Source: Sci Rep. 2021 Apr 5;11:7510. doi: 10.1038/s41598-021-87055-8 (PMC8021558; doi:10.1038/s41598-021-87055-8)
Supplement: Supplementary file 1 — Supplementary Information. [file 41598_2021_87055_MOESM1_ESM.docx]

**Supplementary Figure Legends**

Figure S1. Age distribution of the complete set of patients (A), and of the survived subgroup (B) and the deceased subgroup (C).

Figure S2. Comparative distribution of baseline level (A), differential level (B), medical evaluations (C) parameters in the survived *vs.* deceased subgroups of the ALSS-treated patients. * presented in *log_10_* scale.

Figure S3. Distribution of complications (A) comparative distribution of complications (B) panel pattern of each complication.
