# Supplementary figures and images for: A novel prognostic model to predict outcome of artificial liver support system treatment
Source: Sci Rep. 2021 Apr 5;11:7510. doi: 10.1038/s41598-021-87055-8 (PMC8021558; doi:10.1038/s41598-021-87055-8)

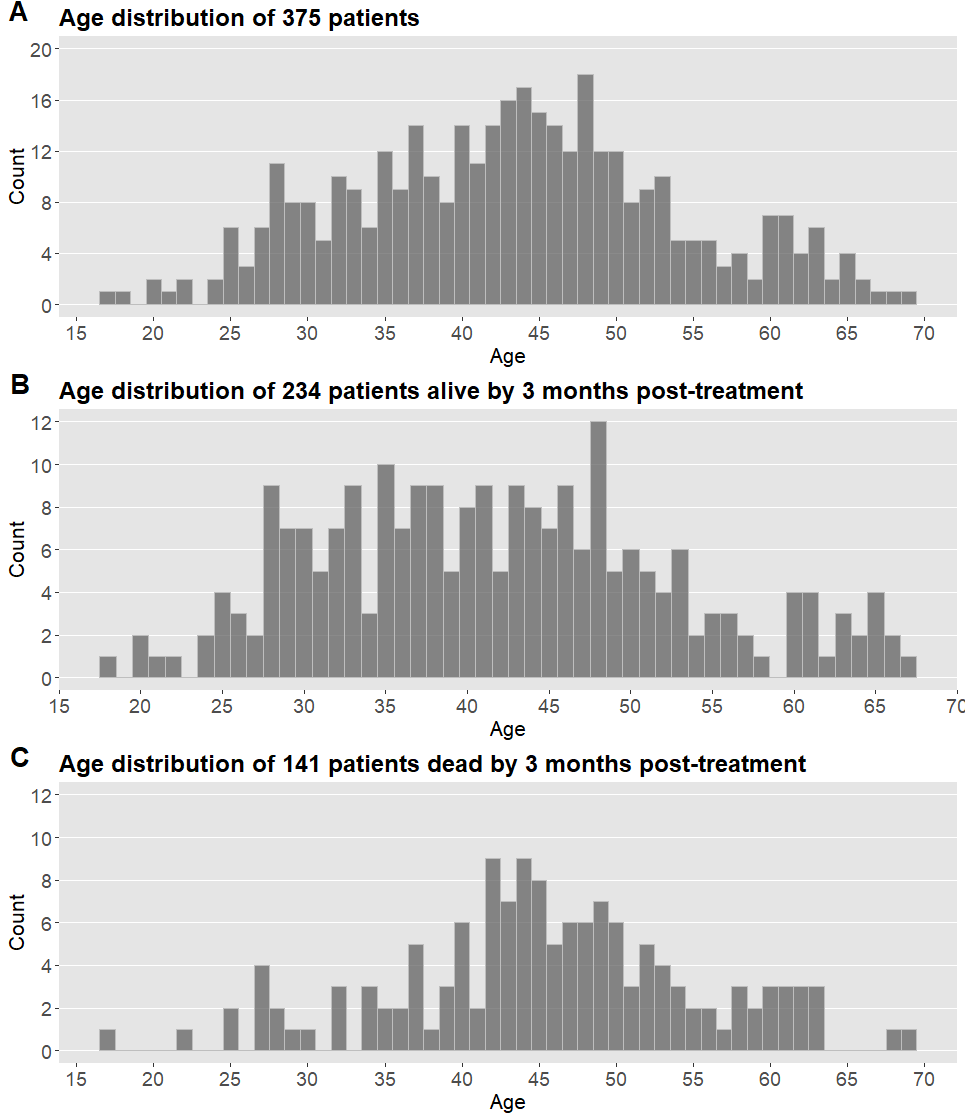

Supplement: Supplementary file 2 — Supplementary Figure S1. [file 41598_2021_87055_MOESM2_ESM.tif]

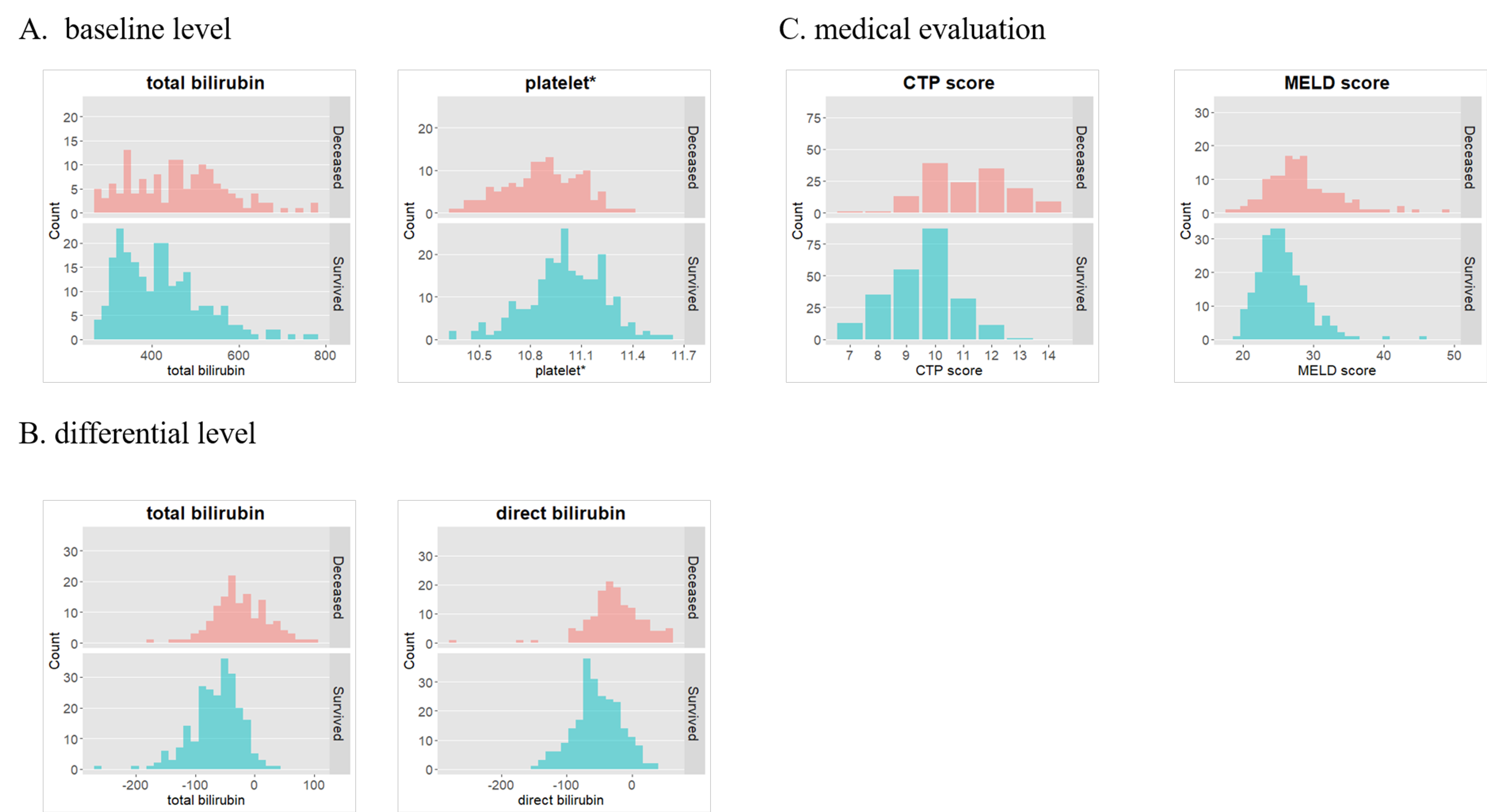

Supplement: Supplementary file 3 — Supplementary Figure S2. [file 41598_2021_87055_MOESM3_ESM.tif]

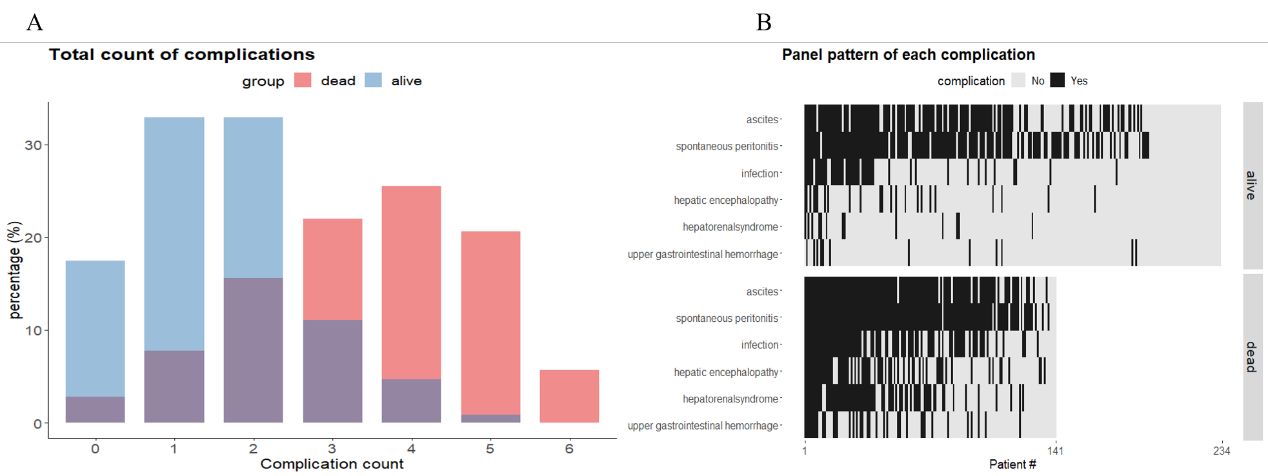

Supplement: Supplementary file 4 — Supplementary Figure S3. [file 41598_2021_87055_MOESM4_ESM.tif]
