# Supplementary material for: A novel prognostic model to predict outcome of artificial liver support system treatment
Source: Sci Rep. 2021 Apr 5;11:7510. doi: 10.1038/s41598-021-87055-8 (PMC8021558; doi:10.1038/s41598-021-87055-8)
Supplement: Supplementary file 5 — Supplementary Table S1. [file 41598_2021_87055_MOESM5_ESM.docx]

**Supplementary Table**

**Table S1. Comparison of parameters in ALSS-treated patients**

|  | | | |
| --- | --- | --- | --- |
|  | alive (n = 234) | dead(n = 141) | *P* |
| ***Baseline level*** |  |  |  |
| HBV-DNA (IU/ml)  CHE (μmol/L) | 39300  4385 | 70500  4271 | 0.72  0.95 |
| total bilirubin (μmol/L) | 408.7 | 461.8 | < 10^-3^ |
| direct bilirubin (μmol/L) | 310.1 | 333.0 | 0.55 |
| ALT (IU/L) | 184 | 173 | 0.35 |
| AST (IU/L) | 150.5 | 163.0 | 0.24 |
| total protein (g/L) | 59.5 | 57.7 | 0.04 |
| albumin (g/L) | 32.7 | 32.4 | 0.19 |
| Na (mmol/L) | 136.6 | 135.5 | 0.02 |
| creatinine (μmol/L) | 77.0 | 84.0 | < 10^-2^ |
| PT | 23.6 | 26.2 | < 10^-3^ |
| INR | 2.0 | 2.3 | < 10^-3^ |
| blood ammonia (μmol/L) | 67.9 | 74.0 | 0.06 |
| platelet | 1.0 * 10^11^ | 7.5 * 10^10^ | < 10^-7^ |
| ***Differenial level***  total bilirubin (μmol/L) | -57.5 | -27.1 | < 10^-14^ |
| direct bilirubin (μmol/L) | -54.0 | -27.7 | < 10^-10^ |
| ALT (IU/L) | -35 | -27 | 0.01 |
| AST (IU/L) | -25.5 | -24.0 | 0.16 |
| total protein (g/L) | -5.7 | -3.6 | < 10^-5^ |
| albumin (g/L) | -1.5 | -1.1 | 0.02 |
| Na (mmol/L) | 2.2 | 1.5 | 0.01 |
| creatinine (μmol/L) | -4.5 | -2.0 | 0.07 |
| PT | -2.1 | -2.3 | 0.42 |
| INR | -0.2 | -0.2 | 0.43 |
| blood ammonia (μmol/L) | -3 | 5 | 0.07 |
| platelet | -1.8 * 10^10^ | -1.0 * 10^10^ | < 10^-4^ |
| recovery percentage  of total bilirubin | 0.600 | 0.700 | 0.21 |
| residual percentage  of total bilirubin  ***Medical evaluation*** | 0.900 | 0.900 | < 10^-22^ |
| CTP at C grade (%) | 56.0 | 89.4 | < 10^-10^ |
| CTP score | 10 | 11 | < 10^-21^ |
| MELD score | 25.2 | 27.6 | < 10^-8^ |
| MELD-Na score | 26.2 | 29.0 | < 10^-7^ |
|  | | | |

HBV - hepatitis B virus; ALT - alanine transaminase; AST - aspartate transaminase; CHE - serum cholinesterase;

PT - prothrombin time; INR - international normalized ratio.
