# Supplementary material for: A novel prognostic model to predict outcome of artificial liver support system treatment
Source: Sci Rep. 2021 Apr 5;11:7510. doi: 10.1038/s41598-021-87055-8 (PMC8021558; doi:10.1038/s41598-021-87055-8)
Supplement: Supplementary file 6 — Supplementary Table S2. [file 41598_2021_87055_MOESM6_ESM.docx]

**Supplementary Table**

**Table S2. Candidate parameters insignificant in univariate logistic regression**

| **parameter** | ***β*** | **95% CI for *β*** | ***P* value** | ***P* < α** | ***P* < α_ad_** |
| --- | --- | --- | --- | --- | --- |
| ***Baseline level*** |  |  |  |  |  |
| log_10_(HBV-DNA) | -0.026 | (-0.175, 0.124) | 0.7372052 | No | No |
| total bilirubin | -0.004 | (-0.006, -0.001) | 0.0022717 | Yes | No |
| direct bilirubin | 0.000 | (-0.003, 0.002) | 0.8146300 | No | No |
| log_10_(ALT) | 0.243 | (-0.308, 0.799) | 0.3882517 | No | No |
| log_10_(AST) | -0.163 | (-0.897, 0.574) | 0.6624423 | No | No |
| total protein | 0.029 | (-0.005, 0.064) | 0.0996039 | No | No |
| albumin | 0.028 | (-0.029, 0.088) | 0.3409641 | No | No |
| Na | 0.068 | (0.012, 0.127) | 0.0205666 | Yes | No |
| PT | -0.050 | (-0.089, -0.014) | 0.0089881 | Yes | No |
| INR | -0.603 | (-1.058, -0.183) | 0.0068840 | Yes | No |
| blood ammonia | -0.008 | (-0.015, 0) | 0.0396938 | Yes | No |
| ***Differential level*** | |  |  |  |  |
| ALT | -0.002 | (-0.005, 0) | 0.1090514 | No | No |
| AST | -0.003 | (-0.007, 0) | 0.1183052 | No | No |
| albumin | -0.089 | (-0.183, 0.003) | 0.0616440 | No | No |
| Na | -0.003 | (-0.045, 0.027) | 0.8609714 | No | No |
| creatinine | -0.024 | (-0.043, -0.009) | 0.0052649 | Yes | No |
| PT | -0.015 | (-0.051, 0.015) | 0.3589378 | No | No |
| INR | -0.156 | (-0.548, 0.018) | 0.3118229 | No | No |
| blood ammonia | -0.006 | (-0.012, 0) | 0.0760047 | No | No |
| platelet | 0.000 | (0, 0) | 0.0026124 | Yes | No |
| recovery percentage of total bilirubin | -0.822 | (-1.826, 0.151) | 0.1014657 | No | No |

Significance level: α = 0.05; α_adj_ = α / 46 = 0.001, adjusting for hypothesis tests of 46 factors.
